# Distinct Phylogeographic Structures of Wild Radish (*Raphanus sativus* L. var. *raphanistroides* Makino) in Japan

**DOI:** 10.1371/journal.pone.0135132

**Published:** 2015-08-06

**Authors:** Qingxiang Han, Hiroyuki Higashi, Yuki Mitsui, Hiroaki Setoguchi

**Affiliations:** 1 Graduate School of Human and Environmental Studies, Kyoto University, Yoshida Nihonmatsu-cho, Sakyo-ku, Kyoto, Japan; 2 Faculty of Agriculture, Tokyo University of Agriculture, Funako 1737, Atsugi, Kanagawa, Japan; Youngstown State University, UNITED STATES

## Abstract

Coastal plants with simple linear distribution ranges along coastlines provide a suitable system for improving our understanding of patterns of intra-specific distributional history and genetic variation. Due to the combination of high seed longevity and high dispersibility of seeds via seawater, we hypothesized that wild radish would poorly represent phylogeographic structure at the local scale. On the other hand, we also hypothesized that wild radish populations might be geographically differentiated, as has been exhibited by their considerable phenotypic variations along the islands of Japan. We conducted nuclear DNA microsatellite loci and chloroplast DNA haplotype analyses for 486 samples and 144 samples, respectively, from 18 populations to investigate the phylogeographic structure of wild radish in Japan. Cluster analysis supported the existence of differential genetic structures between the Ryukyu Islands and mainland Japan populations. A significant strong pattern of isolation by distance and significant evidence of a recent bottleneck were detected. The chloroplast marker analysis resulted in the generation of eight haplotypes, of which two haplotypes (A and B) were broadly distributed in most wild radish populations. High levels of variation in microsatellite loci were identified, whereas cpDNA displayed low levels of genetic diversity within populations. Our results indicate that the Kuroshio Current would have contributed to the sculpting of the phylogeographic structure by shaping genetic gaps between isolated populations. In addition, the Tokara Strait would have created a geographic barrier between the Ryukyu Islands and mainland Japan. Finally, extant habitat disturbances (coastal erosion), migration patterns (linear expansion), and geographic characteristics (small islands and sea currents) have influenced the expansion and historical population dynamics of wild radish. Our study is the first to record the robust phylogeographic structure in wild radish between the Ryukyu Islands and mainland Japan, and might provide new insight into the genetic differentiation of coastal plants across islands.

## Introduction

Coastal plants offer several advantages as a suitable system for improving our understanding of patterns of intra-specific distributional history and genetic variation [1]. First, coastal plants are often widely distributed, covering large geographic ranges latitudinally and longitudinally, often containing both refugial and recolonized areas. Second, they essentially have linear distribution ranges along coastlines, which limit the spatial options for migration and facilitate the reconstruction of distributional limits in the Quaternary glacial period [1, 2]. These advantages characterize coastal plants as a suitable system for inferring distribution history and assessing the effects of historical and extant factors on geographic patterns of genetic variation. Increasing investigations of the geographic distribution of intra-specific genetic variation in a large number of coastal plants have resulted in the detection of the presence or absence of phylogeographic structures at large and local scales.

Investigations of the geographic distribution of intra-specific genetic variation in coastal plants have often resulted in the detection of clear phylogeographic structures; e.g., *Hordeum marinum* [3], *Triglochin maritima* [4], *Eryngium maritimum* [5], and *Carex extensa* [6] in Mediterranean and European coasts, *Zostera marina* in Northern Hemisphere coasts [7], and *Hibiscus tiliaceus* in Pacific and Indian Ocean regions [8]. These patterns were primarily explained by historical processes (e.g., Pleistocene glaciations). Independent colonization was suggested to have occurred in survival populations where suitable habitats were available during Pleistocene glaciations, which in turn influenced the geographic distribution of species by range expansion during the climate amelioration, companied by shaping distinct phylogeographic structures. Some authors have focused on sea currents, which can enable gene flow over a wide range for coastal plants with sea-drifted seeds and constitute a barrier or transport route for seeds or fruits, and thus have strong impacts on geographic patterns of genetic variation [8, 9]. Additionally, some authors have proposed the influence of sea straits as barriers to gene flow contributing to phylogeographic structures [9]. During the last glacial maximum (LGM; ca. 20,000–18,000 BP), the low sea level may have resulted in the drying out of connections between sea basins, restricting dispersal routes of propagules and isolating populations from each other [9]. The absence of phylogeographic structures has rarely been reported [2, 10]. Factors related to specific properties of species, such as clonal growth and/or long-distance dispersal, have been proposed to account for colonization success.

In contrast to the large scale, coastal plants have frequently been reported to lack or have an obscure phylogeographic structure at local scales; e.g., *Calystegia soldanella* (in Europe, Korea, and Japan) [10–12], *Lathyrus japonicus* (Japan) [13], *Suaeda maritima* (Europe) [14], *Uniola paniculata* (Southeastern United States) [15], and *Carex arenaria* (Europe) [16]. The most likely contributing factor was that seeds of coastal plants can be frequently dispersed by sea currents and can survive for long periods [17], which can either erase or prevent the sculpture of the historical phylogeographic structure [10]. However, former studies have reported distinct phylogeographic structures of the coastal plants *Ophiorrhiza japonica* [18] and *Farfugium japonicum* [19] in the Ryukyu Islands. They explained that the past splitting of a land bridge (straits) would have influenced population structures by limiting their geographic range during the Pleistocene climate oscillations.

One coastal plant, wild radish, classified as *Raphanus sativus* L. var. *raphanistroides* Makino (Brassicaceae), is found most commonly on sandy coasts or estuaries and occasionally inland along riverbanks in eastern Asia. Wild radish is characterized by seawater-dispersed seeds surrounded by a water-impermeable seed coat and a large air-filled cavity (spongy pericarp) (S1 Fig). The seeds have been reported to float in seawater and then remain in the seed bank for a prolonged period, during which they are covered by sand, until germination [17]. Thus, wild radish was expected to lack phylogeographic structure as the result of the high potential for frequent seed dispersal at the local scale, such as on the islands of Japan. Indeed, wild radish in Japan was reported to harbor no phylogeographic structure based on allozyme polymorphisms [20], amplification fragment length polymorphism (AFLP) analysis [21], and chloroplast (cp) DNA haplotypes [22]. On the other hand, considerable phenotypic variations among local populations of wild radish in Japan can be identified in our personal observations (S2 Fig). Wild radish plants in the Ryukyu Islands tend to have glabrous leaves and stems, whereas those of mainland Japan are covered by dense setose trichomes. It seems plausible to postulate that the morphological differentiations of wild radish in isolated regions are accompanied by geographical distributions of genetic variation, that is, local populations may be genetically isolated from each other and finally present phylogeographic patterns at a local scale in Japan. Based on this background information, understanding phylogeographic structures would be helpful in providing new insight into the geographical genetic variation of coastal plants on the islands of Japan.

In the present study, we hypothesized the equal possibilities that (a) high frequency gene flow via seed dispersal would decay the phylogeographical structure of the coastal populations on the Japanese islands, or (b) any geographic and/or inorganic factors may sculpt the genetic structure of wild radish, as has been suggested by intra-specific variations of phenotypes. In addition, past population demography, including the traces of bottleneck, range shift histories, and genetic variation of the extant populations, were examined. For these objectives, nuclear DNA microsatellite loci (nuclear simple sequence repeats; nSSRs), and cpDNA haplotypes were used to evaluate the phylogenetic relationships among wild radish populations covering most of the geographic ranges in Japan.

## Materials and Methods

### Sampling and DNA extraction

**Fig 1:**
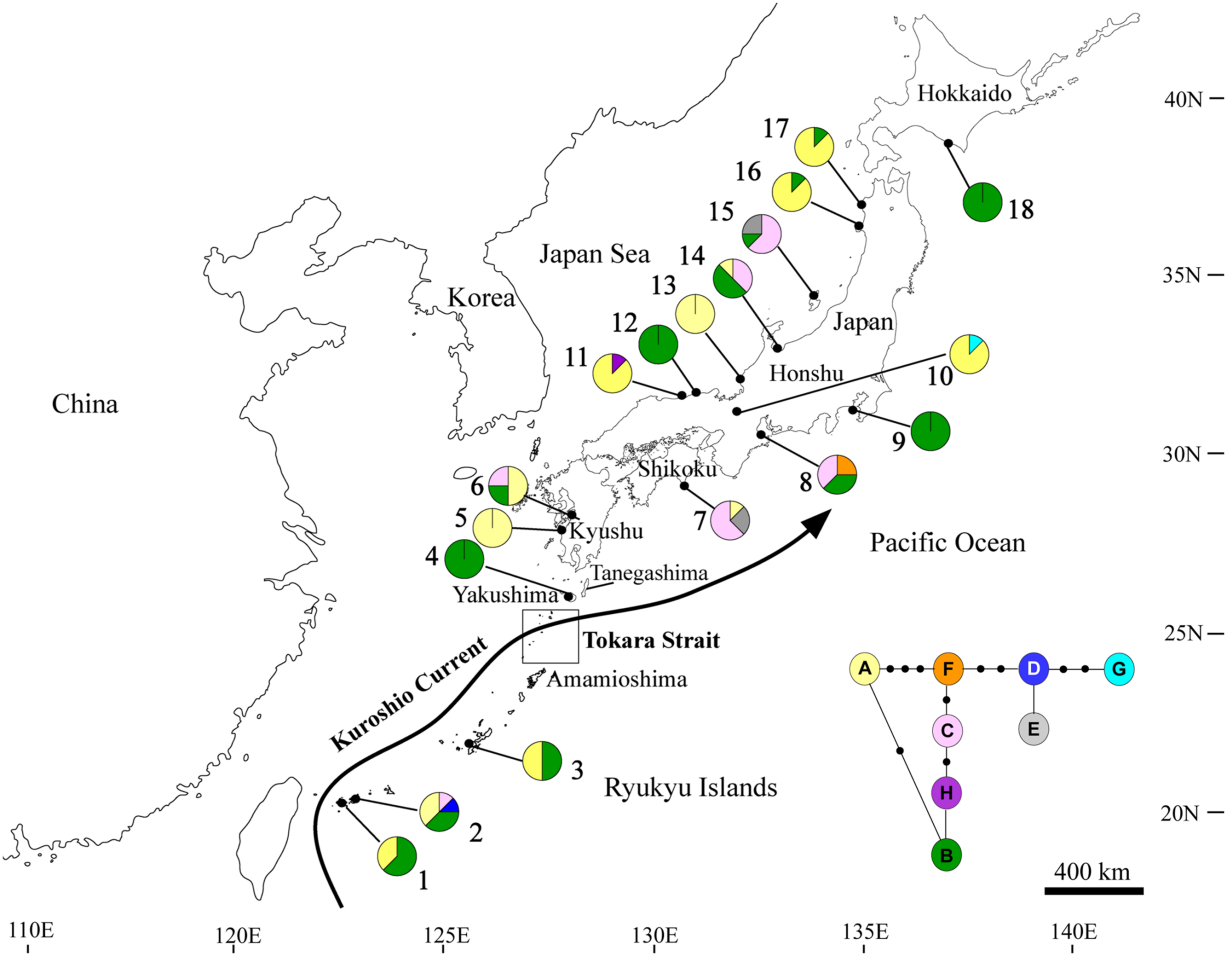
Sampling locations and distribution of chloroplast DNA haplotypes of 18 populations of wild radish sampled in Japan. The pie charts indicate the haplotype frequencies and are color-coded, as for the parsimony network. Population numbers 1–18 correspond to those of Table 1. The square box indicates the Tokara Strait region and is modified from the graphic of Feng et al. [25]. The main path of the Kuroshio Current, modified from the graphic of Yin et al., is also shown [26].

We did not require any specific permission to enter our sampling locations, and we confirmed that our field studies did not compromise endangered or protected species. Leaf material from wild radish was collected from 18 natural populations, covering most of the geographic ranges in Japan, including the Ryukyu Islands, Kyushu Islands, Shikoku Islands, Honshu Islands, and Hokkaido Islands. Details of the locations of populations and the numbers of samples are provided in Fig 1 and Table 1. To avoid sampling close relatives within individual populations, we randomly collected foliar samples at intervals of 10–50 m along the coastlines, with the exception of the population of Shiga (Pop. 10). In this area, the minimum interval was only 3 m because the population was of limited size (as its status was “vulnerable” on the shore of the freshwater Lake Biwa). The sampled leaves were placed immediately in drying silica gel in the field, returned to the laboratory, and stored at 4°C. Dried leaf materials were pulverized into fine powder. Following removal of polysaccharides from the samples using 4-(2-hydroxyethyl)-1-piperazineethanesulfonic acid buffer (HEPES; pH 8.0) [23], DNA was extracted using the cetyltrimethylammonium bromide (CTAB) method [24]. Extracted DNA was dissolved in 100 μL Tris-EDTA (TE) buffer and subjected to amplification by polymerase chain reaction (PCR).

**Table 1:** Sampling localities, sample sizes, and genetic diversity of SSR loci and cpDNA diversity.

No.	Collection site	Sample size	*N*_A_	*H*_O_	*H*_E_	*F*_IS_	Haplotype	*h*	π
	Ryukyu Islands								
1	Iriomote Isl.	26	5.1	0.395 (0.069)	0.566 (0.064)	0.250 (0.101)	AAABBBBB	0.536	0.00195
2	Ishigaki Isl.	27	4.2	0.300 (0.058)	0.565 (0.054)	0.448 (0.093)	AAABBBCD	0.786	0.00291
3	Okinawa Isl.	28	5.1	0.552 (0.067)	0.636 (0.047)	0.147 (0.073)	AAAABBBB	0.571	0.00208
4	Yakushima Isl.	25	3.9	0.498 (0.084)	0.573 (0.054)	0.106 (0.122)	BBBBBBBB	0	0
	Kyushu Island								
5	Kagoshima	25	5.4	0.489 (0.069)	0.661 (0.031)	0.269 (0.090)	AAAAAAA	0	0
6	Kumamoto	29	5.9	0.529 (0.079)	0.672 (0.038)	0.228 (0.096)	AAAABBCC	0.714	0.00343
	Shikoku Island								
7	Kochi	28	4.3	0.377 (0.069)	0.525 (0.053)	0.328 (0.091)	ACCCCCEE	0.607	0.00225
	Honshu Island								
8	Mie	16	4.4	0.500 (0.093)	0.527 (0.072)	0.042 (0.140)	BBBCCCFF	0.750	0.00273
9	Kanagawa	25	5.3	0.458 (0.070)	0.605 (0.060)	0.212 (0.100)	BBBBBBBB	0	0
10	Shiga	55	6.4	0.440 (0.070)	0.624 (0.056)	0.312 (0.067)	AAAAAAAG	0.250	0.00055
11	Tottori	20	4.1	0.406 (0.091)	0.521 (0.079)	0.218 (0.091)	CCCCCCCH	0.250	0.00055
12	Hyogo	29	4.2	0.452 (0.062)	0.569 (0.060)	0.175 (0.090)	BBBBBBBB	0	0
13	Fukui	22	4.7	0.485 (0.110)	0.518 (0.089)	0.173 (0.130)	AAAAAAAA	0	0
14	Toyama	25	5.7	0.511 (0.090)	0.555 (0.074)	0.101 (0.068)	ABBBBCCC	0.679	0.00333
15	Sado Isl.	29	5.3	0.444 (0.095)	0.521 (0.075)	0.217 (0.094)	BCCCCCEE	0.607	0.00225
16	Akita	24	4.1	0.375 (0.082)	0.439 (0.088)	0.123 (0.070)	AAAAAAAB	0.250	0.00091
17	Aomori	29	5.7	0.479 (0.092)	0.526 (0.078)	0.096 (0.087)	AAAAAAAB	0.250	0.00091
	Hokkaido Islands								
18	Hokkaido	24	4.0	0.491 (0.069)	0.598 (0.035)	0.181 (0.111)	BBBBBBBB	0	0
Population Mean		27	4.9	0.454 (0.018)	0.566 (0.015)	0.202 (0.023)		0.347	0.00133

The population numbers are as the same as those used in the following tables and figures; *N*_A_: average number of alleles per site; *H*_E_, expected heterozygosity; *H*_O_, observed heterozygosity; *F*_IS_, inbreeding coefficient; *h*, haplotype diversity; π, nucleotide diversity. Standard errors are given in parentheses.

### Microsatellite genotyping and data analyses

Nine microsatellite markers were selected based on their clarity and reproducibility [27–29] (S1 Table). PCR amplification was performed in a final volume of 5 μL (containing 40–60 ng genomic DNA) following the standard protocol of the Qiagen Multiplex PCR kit (Qiagen, Hilden, Germany). Compound SSR primers [(AC)_6_(AG)_5_, (TC)_6_(AG)_5_, or (GA)_5_(CA)_5_] were labeled with the fluorochrome 6-FAM or HEX (Applied Biosystems, Foster City, CA. USA). Amplification was performed using an initial denaturation step of 15 min at 95°C, followed by 32 cycles of denaturation for 30 s at 95°C, annealing for 90 s at 58°C, and extension for 30 s at 60°C, with a final elongation step at 72°C for 10 min. Amplified products were loaded onto an ABI 3130 autosequencer (Applied Biosystems) using the GeneScan Rox-350 Size Standard (Applied Biosystems) and the POP7 polymer and a 36-cm capillary array, and their sizes were determined using GeneMapper (Applied Biosystems). Departures from Hardy—Weinberg equilibrium (HWE) at each locus were carefully explored using a Markov chain Monte Carlo (MCMC) method implemented in GENEPOP version 4.0.10 [30, 31]. MICRO-CHECKER [32] was employed to estimate the most probable cause of any departure from HWE, including the presence of null alleles, scoring errors caused by stuttering, or allelic dropout attributable to short-allele dominance. Approximately 10% of all samples were amplified and genotyped at least twice; the rate of genotyping error was <5%.

A model-based Bayesian clustering method was applied to assign individuals to populations using the STRUCTURE version 2.3 software [33]. This method identifies *K* (unknown) populations within a dataset and assigns each population/individual to one or more populations/clusters if the individual is admixed. Markov chain Monte Carlo (MCMC) values were set for a burn-in period of 30,000 and a run length of 10^5^ iterations under an admixture model with correlated allele frequencies within populations. The batch run function was carried out for a total of 400 runs (20 runs each for 1–20 clusters; i.e., *K* = 1–20) to quantify the variation of the likelihood of each *K* value. To obtain the correct estimate of the number of clusters, the rate of change of the log probability (*ΔK*) between successive values of *K* was evaluated using the method of Evanno *et al*. [34]. Then, 400 runs of the simulation with the highest modal value of *ΔK* were aligned by the cluster matching and permutation software CLUMPP version 1.1 [35] and presented as bar graphs using DISTRUCT version 1.1 [36].

To assess the genetic diversity of each locus and each population, observed heterozygosity (*H*_O_), Nei’s gene diversity (expected heterozygosity *H*_E_) [37], inbreeding coefficient (*F*_IS_ = 1–*H*_O_/*H*_E_) [38], and population differentiation based on pairwise *F*_ST_ between populations were evaluated using the GENALEX version 6.5 software [39]. The frequency of null alleles (*N*a) [40] was calculated using the formula: (*H*_E_-*H*_O_)/(*H*_E_+*H*_O_) (per locus across all populations). The number of alleles per population was calculated using FSTAT version 2.9.3 [41]. Coefficients of genetic distance (*D*a) [42] were calculated based on pairwise comparisons among the 18 populations of wild radish. A neighbor-joining (NJ) dendrogram was generated to reconstruct phylogenetic relationships among populations using POPULATIONS version 1.2.31 [43]. Bootstrapping based on microsatellite data was conducted using 1000 replicates. To detect recent bottlenecks caused by reductions in effective population size, the observed gene diversity was compared with the equilibrium gene diversity given the observed number of alleles [44, 45] using BOTTLENECK version 1.2.02 [46]. Two models of locus evolution, the infinite allele model (IAM) [47] and the stepwise mutation model (SMM) [48], were used for the analyses together with the sign test [48] and the Bayesian Wilcoxon signed-rank test [49]. The association between pairwise estimates of population differentiation (*F*_ST_/1–*F*_ST_) and the natural logarithm of the corresponding geographic distance was estimated using the Mantel test [50] in GENALEX version 6.5, with significance tested using 1000 permutations.

### Chloroplast DNA haplotyping and data analyses

PCR amplification of cpDNA was conducted for 144 samples (eight individuals per population) using one universal primer pair (*trn*L-F), and two internal primer pairs (*trn*T-L and *rpl*20-*rps*12) designed based on published primers [51, 52] (S2 Table). PCR amplification was conducted in a total reaction volume of 10 μL containing 7.25-μL autoclaved ion-exchanged water, 0.8-μL 2.5 mM dNTP mixture, 1-μL 10× Ex Taq Buffer (Takara Ex Taq; Takara, Kusatsu, Japan), 0.25 U Ex Taq (Takara), 0.2 μM of each primer, and 0.5-μL DNA. Amplification was performed using an initial denaturation step for 5 min at 94°C, followed by 35 cycles of denaturation for 1 min at 94°C, annealing for 1 min at 54°C, and extension for 1.5 min at 72°C. Following amplification, the products were visualized on 0.5%-TAE agarose gels stained with ethidium bromide. PCR products were sequenced using the standard methods of the BigDye Terminator Cycle Sequencing Ready Reaction kit (Applied Biosystems) with the primers specified in S2 Table on an ABI Model 3100 Genetic Analyzer (Applied Biosystems).

All sequence data were analyzed and aligned using Auto Assembler (Applied Biosystems). Basic sequence statistics, including haplotype diversity (*h*) and nucleotide diversity (π), were calculated using the DnaSP version 5.10 software [53]. CpDNA haplotypes were determined based on the aligned sequences, and a parsimony network was constructed using the TCS version 1.21 software [54].

## Results

### Microsatellite diversity and structure

The gene diversity parameters for the nSSRs are shown in Table 1. In total, 102 alleles were detected from the 486 total individuals. The nSSR genetic diversity differed among the 18 populations of wild radish, as indicated by the gene diversity (*H*_E_), ranging from 0.439 (pop. 16) to 0.672 (pop. 6), with an average of 0.566. *H*_O_, which was relatively low compared with *H*_E_, ranging from 0.300 (pop. 2) to 0.522 (pop. 3), with a mean of 0.454. Low rates of inbreeding *F*_IS_ were observed, from 0.042 (pop. 8) to 0.448 (pop. 2), with an average of 0.202. In both the sign and Wilcoxon tests, all populations showed evidence of a recent bottleneck under the assumption of the IAM (S3 Table). With the exception of RsHR026 and RsSA085, the other seven loci exhibited significant deviations from HWE (S1 Table). MICRO-CHECKER analysis suggested that null alleles were present at all nine loci (*P* <0.05), as were scoring errors caused by stuttering (with the exception of RsSA085). However, no evidence of large-scale allele dropout was obtained for any locus (*P* >0.05). The presence of null alleles increases the numbers of homozygotes. Similar degrees of heterozygotic deficiency were evident in Japanese and Korean wild radish populations subjected to analysis of microsatellite [27] and allozyme variations [21]. As the wild radish is self-incompatible, any excess of homozygotes is attributable to the genetic substructure of a local population, in which either the Wahlund effect and/or biparental inbreeding may be in play. Under the SMM, five populations (pops. 4, 7, 11, 12, and 16) exhibited significant heterozygosity excess (i.e., evidence of a recent bottleneck) by the sign test and nine populations (pops. 2, 3, 4, 7, 11, 12, 13, 16, and 18) by the Wilcoxon test (S3 Table). A highly positive relationship was observed between pairwise genetic distance and geographic distance over total populations (*R* ^2^ = 0.2953, *p*<0.001; S3 Fig), suggesting a significant isolation by distance (IBD) pattern across all 18 wild radish populations in Japan.

**Fig 2:**
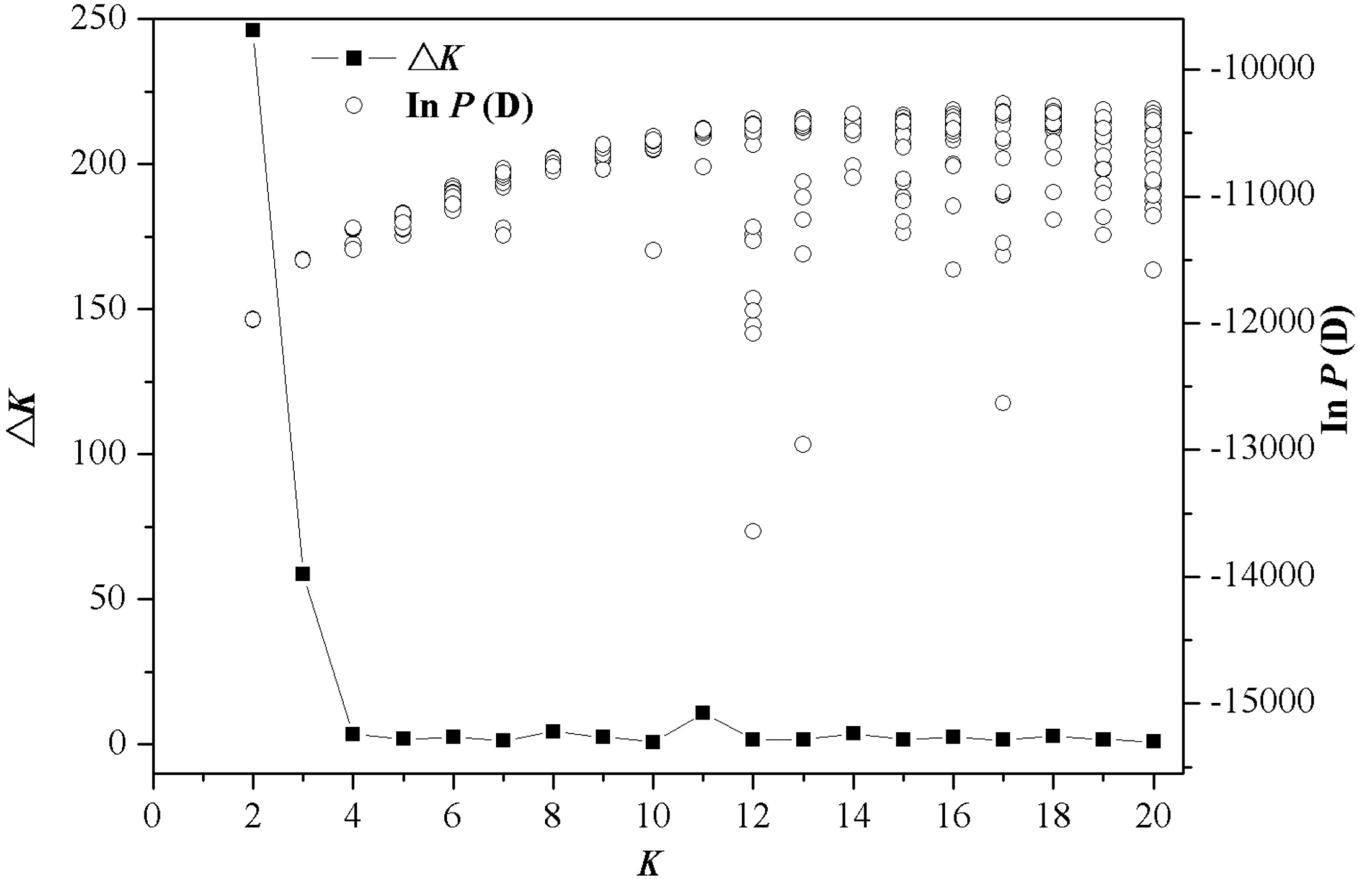
Structure analysis of SSR data from wild radish populations distributed across Japan. The Δ*K* values are shown, as are the mean posterior probability values [the Ln *P*(D) values] (one for each *K*).

**Fig 3:**
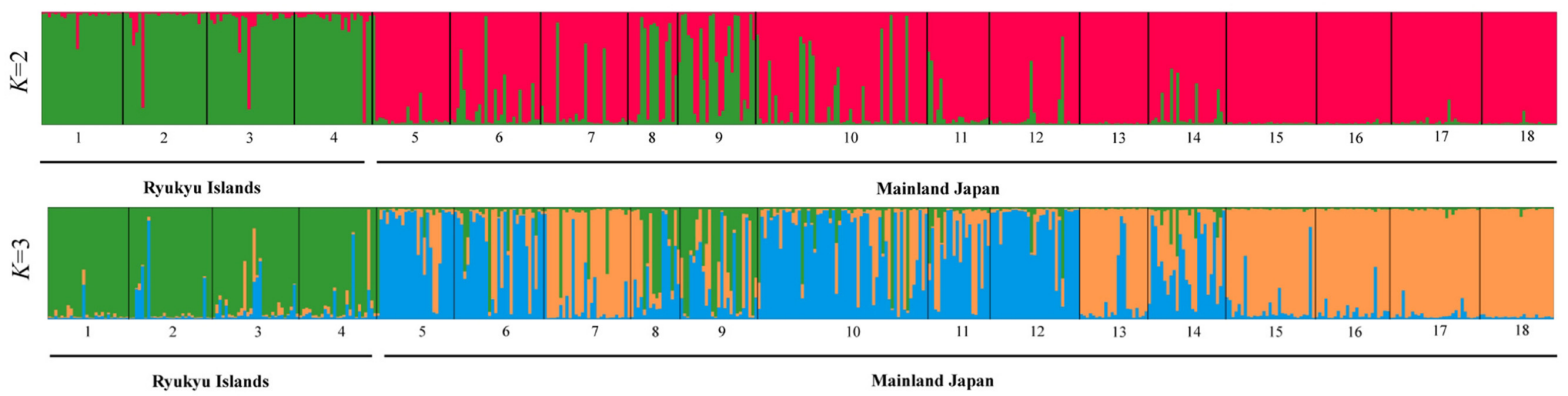
Individual assignment via STRUCTURE analysis using models with *K* = 2 and *K* = 3. The numbers in each population are shown beneath the bars. The numbers in the 18 populations (486 individuals in total) are the same as those in Fig 1 and Table 1.

The results of Bayesian clustering analysis indicated that the most likely number of clusters was 2 when the *ΔK* statistics were applied (*ΔK =* 2; Fig 2), which was strongly supported by the highest similarity coefficient values (*H*) using CLUMPP analysis (S4 Fig). The cluster analysis revealed that the Ryukyu Islands populations (pops. 1–4) and mainland populations (pops. 5–18) were genetically distinct (Fig 3). In addition, the similarity coefficient *H* determined using CLUMPP suggested the other appropriate number of clusters to be *K* = 3, with the second highest *ΔK* statistics. Thus, the Ryukyu Islands populations were clearly assigned to independent clusters using the two most appropriate numbers of clusters. The alpha values (alpha is the Dirichlet parameter evaluating the extent of admixture) varied greatly throughout the runs (*K* = 2 and *K* = 3) (S5 and S6 Figs), indicating that the [Ln *P*(D)] values had been accurately estimated for each *K* [55]. In accordance with the results of Bayesian clustering analysis, two major clusters were identified from NJ phylogeny (S7 Fig) and weakly supported by bootstrapping (58%); one group comprised the Ryukyu Islands populations (pops. 1–4) and the other comprised the mainland Japan populations (pops. 5–18).

### Chloroplast DNA diversity and haplotype distribution

Based on approximately 1,375 bp of the three noncoding regions of cpDNA, eight haplotypes were detected in a total of 144 individuals from 18 wild radish populations. The haplotype diversity (*h*) and nucleotide diversity (π) of cpDNA sequences are shown in Table 1. The haplotype diversity (*h*) ranged from 0 to 0.786, with an average of 0.347. The nucleotide diversity (π) varied from 0 to 0.343, with an average of 0.00133.

The distribution of the observed eight haplotypes of wild radish is shown as a parsimony network in Fig 1. A cpDNA haplotype network is created by homoplasy that is, in turn, caused by recurrent mutations at the same sites [56] and/or possible recombinations (that have previously been detected in radish cpDNA; [57]). Haplotypes B and A were broadly distributed in wild radish. Haplotype B was the most abundant (38.9%) and widely distributed across the Japanese Archipelago. Haplotype A also ranged across the archipelago (36.8%), but at a lower frequency than that of haplotype B. Specifically, only haplotype D was restricted to Ishigaki Island (pop. 2) of the Ryukyu Islands, whereas haplotypes E, F, G, and H were unique to the mainland populations. Of the 18 populations surveyed, six were dominated by single haplotypes (A and B). With the exception of pop. 2, which exhibited four haplotypes, the Ryukyu Island populations (pops. 1, 3, and 4) were fixed with haplotypes A and/or B, identical to the majority of wild radish populations in mainland Japan.

## Discussion

### Population structure

The present study provides the first evidence of clear geographic structure observed in coastal plants between the Ryukyu Islands and the adjacent mainland Japan; wild radish populations in northern and southern Japan were clearly demarcated around Yakushima Island (the northernmost area of Ryukyu Islands) based on microsatellite variation. This distinct phylogeographic structure is likely attributable to two major causes. First, sea currents are proposed to influence the phylogeographic structure of wild radish in Japan by shaping barriers and transport of seed dispersal. The present Kuroshio Current originates from the North Equatorial Current in the West Pacific, and flows along the Ryukyu Islands towards the north. Then it moves eastward, finally reaching the southern sea of the main islands of Japan through the Tokara Strait near Yakushima Island (Fig 1) [58, 59]. Considering the high dispersibility of seeds via seawater in coastal plants, the Kuroshio Current is proposed to play two contrasting roles on the phylogeographic structure of wild radish. On one hand, the Kuroshio Current promotes gene exchanges among the isolated Ryukyu Islands by transporting spongy fruits of wild radish, and induces uniformity in their genetic structure across the Ryukyu Islands, which stretch over 1,000 km. On the other hand, it also acts as a geographic barrier against seed dispersal and in turn shapes the genetic gap between the Ryukyu Islands and mainland Japan.

The Kuroshio Current effect is also likely responsible for the genetic admixtures between several mainland populations (pops. 8 and 9) and Ryukyu Islands populations. Pops. 8 (in Mie) and 9 (in Kanagawa) are greatly distant from the Ryukyu Islands but are located downstream of the Kuroshio Current, which flows from Ryukyu Islands areas. Accordingly, seeds of wild radish are unavoidably and limitedly transported from the Ryukyu Islands to these populations, promoting gene exchange between these regions. This finding coincides with a previous study that reported genetic similarity in wild radish among one insular population of the Ryukyu Islands and several populations from the western part of the Pacific coastal area (near Mie prefecture) based on the allozyme diversity [20]. In general, sea currents strongly affect geographic genetic structure in coastal species by constituting a barrier or transport mechanism for seeds or fruits [1, 8, 12, 60]. Distinctly and originally, the present study recorded the two contrasting effects of the Kuroshio Current functioning simultaneously on the coastal plants, which might provide new insight into genetic differentiation across the islands of Japan. On the other hand, phenotypes of the wild radish (trichomes and length of hypocotyl) in the western part of the Pacific coastal area are apparently the same as those of mainland Japan wild radish (dense setose in leaves and stems) (personal observation), suggesting that seed migration and settlement are rare.

Second, the Tokara Strait was thought to have created a boundary between the Ryukyu Islands and mainland Japan. The Tokara Strait is located between Amamioshima and Tanegashima Islands, south of Kyushu (Fig 1) [25], and has persisted since its formation in the Pliocene, ca. 2–5 Ma [61]. During the glacial period, ocean water levels are estimated to have been more than 100 m lower. However, the Tokara Strait still existed because the water depth was more than 1,000 m. As a result, the migration of species north and south of the Tokara Strait is assumed to have been blocked, and thus it formed a borderline [18, 62]. Therefore, the Tokara Strait can be assumed to have been a geographic barrier, which may ultimately have resulted in the genetic divergence between the Ryukyu Islands and mainland Japan populations of wild radish. Considering the splitting of the land bridge during the LGM period, plants in both the Ryukyu Islands and mainland Japan were supposed to have experienced different migration and recolonization histories [63], and in turn, vicariance events likely occurred. Thus, it is supposed that the Tokara Strait had a significant influence on the distribution of wild radish, shaping the geographic distribution of genetic variation in the Ryukyu Islands and mainland Japan. In addition, the Tokara Strait is the main pathway of the Kuroshio Current when it moves eastward, thus the Tokara Strait and Kuroshio Current work together to shape a robust barrier against migration between the Ryukyu Islands and mainland Japan.

In contrast to the clear phylogeographic structure detected by microsatellite loci, no geographic distribution pattern of haplotypes was observed in cpDNA variations. The discordance is often associated with the different inheritance modes and evolutionary rates between nuclear and chloroplast genomes. Nuclear genes are biparentally inherited and dispersed, whereas the chloroplast genes are inherited predominantly or entirely from the female parent. They segregate rapidly during vegetative growth, so the low heterozygosity in chloroplast genes is feasibly shaped without recombination [64]. This would also be the contributing factor for the low levels of cpDNA variation in terms of nucleotide and haplotype diversity within populations of wild radish (Table 1). The other possible main reason would be the low evolutionary rate of cpDNA. Previous studies that compared the rate of substitution both at synonymous sites and in noncoding sequences have suggested that chloroplast genes have lower rates of nucleotide substitution (only half of the nuclear DNA evolutionary rate) [65]. Accordingly, cpDNA variation infers a past change in population demographics, such as population expansion or decline, whereas microsatellite variation reflects recent events in the population. Thus, the geographic distribution of cpDNA variation in wild radish in isolated Ryukyu Islands and mainland Japan is assumed to have required time to become established.

### Population demography

Evidence that most populations shared the common cpDNA haplotypes (A and B) across the entire Japanese archipelago (Fig 1) suggests a scenario in which wild radish along the archipelago might have derived from a common ancestor, that is, the coalescent effect, which leads to the wide geographic distribution of ancestral haplotypes [66, 67]. Namely, the sharing of the predominant haplotypes among all populations may be most plausibly explained by the retention of the ancestral haplotypes. Due to the considerable genetic drift in wild radish populations, some haplotypes have randomly diminished, which likely resulted in some haplotypes being unique to particular populations (e.g., haplotype D was unique to pop. 3). Other possibilities should also be considered: gene flow is likely responsible for the haplotypes common to the total population. A combination of the high frequency of long-distance dispersal via ocean currents and the high longevity of seeds could eventually result in hybridization and/or introgression, although phenotypic variation may depress the reproductive success of immigrants to some extent.

Historical population dynamics of wild radish were reflected by the significant bottleneck effect and IBD pattern. The recent bottleneck in wild radish populations on the islands of Japan is most plausibly explained by frequent submergence caused by the high frequency of habitat disturbances, such as frequent intermittent typhoons, striking wild radish populations. In addition, due to the relatively small geographic size of islands and the past fragmentation during the LGM period, plants in islands show increased sensitivity to genetic drift. The significant correlation between geographical and genetic distances indicates the model of stepwise range expansion in wild radish. It is attributed to the linear arrangement of habitats, with seeds assumed to move in a linear pattern via sea currents, that is, geographically close populations are phylogenetically close. Due to the genetic boundary between the Ryukyu Islands and mainland Japan, migration is restricted or rare, but not impossible, as evidenced from the long floating time and long viability of seeds. In addition, the numerous multidirectional tributaries of sea currents surrounding the Japanese archipelago are also responsible for the expansion of wild radish populations. Our results are consistent with the positive correlation of IBD in wild radish reported by Ohsako et al. [27] using microsatellite loci, as well as in other coastal plants such as *Cakile maritima*, *Salsola kali*, and *Halimione portulacoides* in the Atlantic clusters [1].

#### Environment factors

Fruits of wild radish comprise a few-seeded capsule with a very solid and water impermeable seed coat containing a large air-filled cavity; these characteristics are essential to enhancing seed buoyancy, promoting seed dispersal by seawater over long distances, and high longevity. Accordingly, sea currents enable frequent gene flow over the wide distribution range of wild radish. However, the Ryukyu Islands and mainland Japan experience different histories and hence produce different selection pressures, and these in turn shape the genetic heterogeneity between local populations by producing different selective forces. Also, these two geographic ranges differ in both abiotic (e.g., temperature and precipitation) and biotic (such as the extensive morphological variation of wild radish) factors, which may generate ecological barriers against gene flow. These two forces work synergistically, producing genetic heterogeneity in natural populations and thus enhance genetic differentiation and preserve the robust phylogeographic structure. This hypothesis does not conflict with the observation of genetic admixture between several mainland populations (pops. 8 and 9) and the Ryukyu Islands, which exhibit great morphological variation, implying that restricted gene flow could not be disturbed by the existing robust geographic pattern of genetic variation. However, abiotic factors (such as vicariance mediated by sea currents) and/or *in situ* factors could not be determined to be causative, thus it is necessary to perform studies involving reciprocal transplantation and/or sympatric cultivation of wild accessions.

## Conclusions

Considering wild radish as a typical coastal plant with high longevity and high dispersibility of seeds via sea currents, it poorly represents phylogeographic structure at local scales. However, our study is the first to record the robust phylogeographic structure in wild radish between the Ryukyu Islands and the adjacent mainland Japan, which might provide new insight into the genetic differentiation of coastal plants across islands. The Kuroshio Current has an important influence on the geographic distribution of genetic variation of wild radish by shaping the genetic gap between isolated populations. In addition, the Tokara Strait is proposed to have been responsible for the genetic isolation between these two clusters. Finally, extant habitat disturbances (coastal erosion), migration patterns (linear expansion), and geographic characteristics (small islands and surrounding sea currents) have influenced the expansion and historical population dynamics of wild radish.

## Supporting Information

S1 Fig Capsules and seeds of wild radish. Fruits of wild radish are capsules (left), however, its spongy pericarp is indehiscent and separate at each locule. Seawater dispersal is usually accomplished in this unit (middle). One seed is enveloped by spongy pericarp in each locule (right).
(TIF)

S2 Fig Phenotypic variations within wild radish. (a) Leaf of wild radish from the Ryukyu Islands; (b) Leaf of wild radish from mainland Japan; (c) Stem of wild radish from the Ryukyu Islands; (d) Stem of wild radish from mainland Japan. Wild radish were planted under identical cultivation conditions (with and without vernalization) in a greenhouse (21°C).
(TIF)

S3 Fig Pairwise population differentiation (*F*_ST_/1–*F*_ST_) values and the natural logarithms of the corresponding geographic distances among the 18 wild radish populations. (TIF)

S4 Fig Similarity coefficient *H*, estimated using the CLUMPP software. (TIF)

S5 Fig Histograms of the alpha values obtained throughout the run with *K* = 2 according to Structure Analysis. (TIF)

S6 Fig Histograms of the alpha values obtained throughout the run with *K* = 3 according to Structure Analysis. (TIF)

S7 Fig Neighbor-joining phylogenetic tree of the 18 wild radish populations based on genetic distances (*D*a) among populations. (TIF)

S1 Table Characteristics of nine polymorphic microsatellite primers used. (DOCX)

S2 Table Information of three cpDNA primer pairs. (DOCX)

S3 Table Probability of a Bottleneck effect for each of the 18 populations of wild radish. (DOCX)

## References

[1] Kadereit JW, Arafeh R, Somogyi G, Westberg E. Terrestrial growth and marine dispersal? Comparative phylogeography of five coastal plant species at a European scale. Taxon. 2005; 54: 861–876.

[2] Kadereit JW, Westberg E. Determinants of phylogeographic structure: a comparative study of seven coastal flowering plant species across their European range. Watsonia. 2007; 26: 229–238.

[3] Jakob SS, Ihlow A, Blattner FR. Combined ecological niche modelling and molecular phylogeography revealed the evolutionary history of *Hordeum marinum* (Poaceae)—niche differentiation, loss of genetic diversity, and speciation in Mediterranean Quaternary refugia. Mol Ecol. 2007; 16: 1713–1727. 17402985 10.1111/j.1365-294X.2007.03228.x

[4] Lambracht E, Westberg E, Kadereit JW. Phylogeographic evidence for the postglacial colonization of the North and Baltic Sea coasts from inland glacial refugia by *Triglochin maritima* L. Flora. 2007; 202: 79–88.

[5] Clausing G, Vickers K, Kadereit JW. Historical biogeography in a linear system: genetic variation of Sea Rocket (*Cakile maritima*) and Sea Holly (*Eryngium maritimum*) along European coasts. Mol Ecol. 2000; 9: 1823–1833. 11091318 10.1046/j.1365-294x.2000.01083.x

[6] Escudero M, Vargas P, Arens P, Ouborg NJ, Luceño M. The east-west-north colonization history of the Mediterranean and Europe by the coastal plant *Carex extensa* (Cyperaceae). Mol Ecol. 2010; 19: 352–370. 10.1111/j.1365-294X.2009.04449.x 20002603

[7] Olsen JL, Stam WT, Coyer JA, Reusch TB, Billingham M, Boström C, et al North Atlantic phylogeography and large-scale population differentiation of the seagrass *Zostera marina* L. Mol Ecol. 2004; 13: 1923–1941. 15189214 10.1111/j.1365-294X.2004.02205.x

[8] Takayama K, Tateishi Y, Murata J, Kajita T. Gene flow and population subdivision in a pantropical plant with sea-drifted seeds *Hibiscus tiliaceus* and its allied species: evidence from microsatellite analyses. Mol Ecol. 2008; 17: 2730–2742. 10.1111/j.1365-294X.2008.03799.x 18482261

[9] Westberg E, Kadereit JW. The influence of sea currents, past disruption of gene flow and species biology on the phylogeographical structure of coastal flowering plants. J Biogeogra. 2009; 36: 1398–1410.

[10] Arafeh R, Kadereit JW. Long-distance seed dispersal, clone longevity and lack of phylogeographical structure in the European distributional range of the coastal *Calystegia soldanella* (L.) R. Br.(Convolvulaceae). J Biogeogra 2006; 33: 1461–1469.

[11] Kim ST, Chung MG. Genetic and clonal diversity in Korean populations of *Calystegia soldanella* (Convolvulaceae). Isr J Plant Sci. 1995; 43: 213–226.

[12] Noda A, Mitsui Y, Ikeda H, Setoguchi H. Long-term isolation of the coastal plant *Calystegia soldanella* (Convolvulaceae) in ancient freshwater Lake Biwa, Japan. Biol J Linn Soc. 2011; 102: 51–66.

[13] Ohtsuki T, Kaneko Y, Setoguchi H. Isolated history of the coastal plant *Lathyrus japonicus* (Leguminosae) in Lake Biwa, an ancient freshwater lake. AoB plants 2011: plr021 10.1093/aobpla/plr021 22476491 PMC3176521

[14] Weising K, Freitag H. Phylogeography of halophytes from European coastal and inland habitats. Zool Anz. 2007; 246: 279–292.

[15] Franks SJ, Richards CL, Gonzales E, Cousins J, Hamrick J. Multi-scale genetic analysis of *Uniola paniculata* (Poaceae): a coastal species with a linear, fragmented distribution. Am J Bot. 2004; 91: 1345–1351. 10.3732/ajb.91.9.1345 21652367

[16] Jonsson BO, Prentice HC. Allozyme diversity and geographic variation in the widespread coastal sedge, *Carex arenaria* . Divers Distrib. 2000; 6: 65–80.

[17] Ridley HN. Dispersal of plants throughout the world; 1930.

[18] Nakamura K, Denda T, Kokubugata G, Suwa R, Yang T, Peng CI, et al Phylogeography of *Ophiorrhiza japonica* (Rubiaceae) in continental islands, the Ryukyu Archipelago, Japan. J Biogeogra. 2010; 37: 1907–1918.

[19] Seo A, Watanabe M, Hotta M, Murakami N. Geographical patterns of allozyme variation in *Angelica japonica* (Umbelliferae) and *Farfugium japonicum* (Compositae) on the Ryukyu Islands, Japan. APG: Acta Phytotax et Geobot. 2004; 55: 29–44.

[20] Huh MK, Ohnishi O. Allozyme diversity and population structure of Japanese and Korean populations of wild radish, *Raphanus sativus* var. *hortensis* f. *raphanistroides* (Brassicaceae). Genes Genet Syst. 2001; 76: 15–23. 11376547 10.1266/ggs.76.15

[21] Huh MK, Ohnishi O. Genetic Diversity and Genetic Relationships of East Asian Natural Populations of Wild Radish Revealed by AFLP. Breeding Sci. 2002; 52: 79–88.

[22] Ohsako T, Ohnishi O. Chloroplast DNA variability in Japanese and Korean populations of wild radishes *Raphanus sativus* var. *hortensis* f. *raphanistroides* (Brassicaceae). Scientific Reports of Kyoto Prefectural University Human Environment and Agriculture (Japan) 2007.

[23] Setoguchi H, Ohba H. Phylogenetic relationships in *Crossostylis* (Rhizophoraceae) inferred from restriction site variation of chloroplast DNA. J Plant Res. 1995; 108: 87–92.

[24] Doyle J, Doyle J. Isolation of DNA from small amounts of plant tissues. Focus. 1990; 12: 13–15.

[25] Feng M, Mitsudera H, Yoshikawa Y. Structure and Variability of the Kuroshio Current in Tokara Strait. J Phys Oceanogr. 2000; 30: 2257–2276.

[26] Yin W, Fu C, Guo L, He Q, Li J, Jin B, et al Species delimitation and historical biogeography in the genus *Helice* (Brachyura: Varunidae) in the Northwestern Pacific. Zool Sci. 2009; 26: 467–475. 10.2108/zsj.26.467 19663641

[27] Ohsako T, Hirai M, Yamabuki M. Spatial structure of microsatellite variability within and among populations of wild radish *Raphanus sativus* L. var. *hortensis Backer* f. *raphanistroides* Makino (Brassicaceae) in Japan. Breeding Sci. 2010; 60: 195–202.

[28] Hashida T, Nakatsuji R, Budahn H, Schrader O, Peterka H, Fujimura T, et al Construction of a chromosome-assigned, sequence-tagged linkage map for the radish, *Raphanus sativus* L. and QTL analysis of morphological traits. Breeding Sci. 2013; 63: 218–226. 10.1270/jsbbs.63.218 PMC3688384 23853517

[29] Nakatsuji R, Hashida T, Matsumoto N, Tsuro M, Kubo N, Hirai M. Development of genomic and EST-SSR markers in radish (*Raphanus sativus* L.). Breeding Sci. 2011; 61: 413–419. 10.1270/jsbbs.61.413 PMC3406777 23136479

[30] Raymond M, Rousset F. GENEPOP (version 1.2): population genetics software for exact tests and ecumenicism. J Hered. 1995; 86: 248–249.

[31] Rousset F. genepop’007: a complete re-implementation of the genepop software for Windows and Linux. Mol Ecol Resour. 2008; 8: 103–106. 10.1111/j.1471-8286.2007.01931.x 21585727

[32] Van Oosterhout C, Hutchinson WF, Wills DP, Shipley P. MICRO-CHECKER: software for identifying and correcting genotyping errors in microsatellite data. Mol Ecol Notes. 2004; 4: 535–538.

[33] Pritchard JK, Stephens M, Donnelly P. Inference of population structure using multilocus genotype data. Genetics. 2000; 155: 945–959. 10835412 10.1093/genetics/155.2.945 PMC1461096

[34] Evanno G, Regnaut S, Goudet J. Detecting the number of clusters of individuals using the software STRUCTURE: a simulation study. Mol Ecol. 2005; 14: 2611–2620. 15969739 10.1111/j.1365-294X.2005.02553.x

[35] Jakobsson M, Rosenberg NA. CLUMPP: a cluster matching and permutation program for dealing with label switching and multimodality in analysis of population structure. Bioinformatics. 2007; 23: 1801–1806. 17485429 10.1093/bioinformatics/btm233

[36] Rosenberg NA. DISTRUCT: a program for the graphical display of population structure. Mol Ecol Notes. 2004; 4: 137–138.

[37] Nei M. Molecular evolutionary genetics: Columbia University Press; 1987.

[38] Nei M. Estimation of average heterozygosity and genetic distance from a small number of individuals. Genetics. 1978; 89: 583–590. 17248844 10.1093/genetics/89.3.583 PMC1213855

[39] Peakall R, Smouse PE. GenAlEx 6.5: genetic analysis in Excel. Population genetic software for teaching and research-an update. Bioinformatics. 2012; 28: 2537–2539. 22820204 10.1093/bioinformatics/bts460 PMC3463245

[40] Brookfield J. A simple new method for estimating null allele frequency from heterozygote deficiency. Mol Ecol. 1996; 5: 453–455. 8688964 10.1111/j.1365-294x.1996.tb00336.x

[41] Goudet J. FSTAT, a program to estimate and test gene diversities and fixation indices (version 2.9. 3); 2001.

[42] Nei M, Tajima F, Tateno Y. Accuracy of estimated phylogenetic trees from molecular data. J Mol Evol. 1983; 19: 153–170. 6571220 10.1007/BF02300753

[43] Langella O. Populations (Logiciel de genétique des populations). CNRS, France Available: http://bioinformaticsorg/~tryphon/populations/; 2000.

[44] Watterson G. The homozygosity test of neutrality. Genetics. 1978; 88: 405–417. 17248803 10.1093/genetics/88.2.405 PMC1213809

[45] Watterson G. The homozygosity test after a change in population size. Genetics. 1986; 112: 899–907. 3957010 10.1093/genetics/112.4.899 PMC1202784

[46] Piry S LG, Cornuet JM. Bottleneck: a computer program for detecting recent reductions in effective population size from allele frequency data. J Hered. 1999; 90: 502–503.

[47] Maruyama T, Fuerst PA. Population bottlenecks and nonequilibrium models in population genetics. II. Number of alleles in a small population that was formed by a recent bottleneck. Genetics. 1985; 111: 675–689. 4054612 10.1093/genetics/111.3.675 PMC1202664

[48] Cornuet JM, Luikart G. Description and power analysis of two tests for detecting recent population bottlenecks from allele frequency data. Genetics. 1996; 144: 2001–2014. 8978083 10.1093/genetics/144.4.2001 PMC1207747

[49] Luikart G, Allendorf FW, Cornuet JM, Sherwin WB. Distortion of allele frequency distributions provides a test for recent population bottlenecks. J Hered. 1998; 89: 238–247. 9656466 10.1093/jhered/89.3.238

[50] Mantel N. The detection of disease clustering and a generalized regression approach. Cancer Res. 1967; 27: 209–220. 6018555

[51] Taberlet P, Gielly L, Pautou G, Bouvet J. Universal primers for amplification of three non-coding regions of chloroplast DNA. Plant Mol Biol. 1991; 17: 1105–1109. 1932684 10.1007/BF00037152

[52] Hamilton M. Four primer pairs for the amplification of chloroplast intergenic regions with intraspecific variation. Mol Ecol. 1999; 8: 521–523. 10199016

[53] Librado P, Rozas J. DnaSP v5: a software for comprehensive analysis of DNA polymorphism data. Bioinformatics. 2009; 25: 1451–1452. 10.1093/bioinformatics/btp187 19346325

[54] Clement M, Posada D, Crandall KA. TCS: a computer program to estimate gene genealogies. Mol Ecol. 2000; 9: 1657–1659. 11050560 10.1046/j.1365-294x.2000.01020.x

[55] Pritchard JK, Wen W, Falush D. Documentation for structure software: version 2. 2003.

[56] Posada D, Crandall KA. Intraspecific gene genealogies: trees grafting into networks. Trends Ecol Evol. 2001; 16: 37–45. 11146143 10.1016/s0169-5347(00)02026-7

[57] Yamagishi H, Glimelius K. Somatic hybrids between Arabidopsis thaliana and cytoplasmic male-sterile radish (*Raphanus sativus*). Plant Cell Rep. 2003; 22: 52–58. 12827437 10.1007/s00299-003-0655-0

[58] Tanaka Y, Ono T. Late Quarternary paleoceanographic record from the middle Ryukyu Trench slope, northwest Pacific. Mar Micropaleontol. 1991; 18: 115–128.

[59] Ujiie H, Ujiie Y. Late Quaternary course changes of the Kuroshio Current in the Ryukyu Arc region, northwestern Pacific Ocean. Mar Micropaleontol. 1999; 37: 23–40.

[60] Bond J, Daniels R, Bioret F. Genetic diversity in Crambe maritima along the English Channel: the role of ocean currents in determining population structure. Ecography. 2005; 28: 374–384.

[61] Ota H. Geographic patterns of endemism and speciation in amphibians and reptiles of the Ryukyu Archipelago, Japan, with special reference to their paleogeographical implications. Res Popul Ecol. 1998; 40: 189–204.

[62] Kubota Y, Hirao T, Fujii Sj, Shiono T, Kusumoto B. Beta diversity of woody plants in the Japanese archipelago: the roles of geohistorical and ecological processes. J Biogeogr. 2014; 41: 1267–1276.

[63] Okaura T, Quang ND, Ubukata M, Harada K. Phylogeographic structure and late Quaternary population history of the Japanese oak *Quercus mongolica* var. *crispula* and related species revealed by chloroplast DNA variation. Genes Genet Syst. 2007; 82: 465–477. 18270437 10.1266/ggs.82.465

[64] Banks JA, Birky CW. Chloroplast DNA diversity is low in a wild plant, Lupinus texensis. Proc Natl Acad Sci. 1985; 82: 6950–6954. 2995994 10.1073/pnas.82.20.6950 PMC391287

[65] Wolfe KH, Li WH, Sharp PM. Rates of nucleotide substitution vary greatly among plant mitochondrial, chloroplast, and nuclear DNAs. Proc Natl Acad Sci. 1987; 84: 9054–9058. 3480529 10.1073/pnas.84.24.9054 PMC299690

[66] Crandall KA, Templeton AR. Empirical tests of some predictions from coalescent theory with applications to intraspecific phylogeny reconstruction. Genetics. 1993;134: 959–969. 8349118 10.1093/genetics/134.3.959 PMC1205530

[67] Nordborg M. Coalescent theory. Handbook of statistical genetics 2001.

