# Supplementary figures and images for: Distinct Phylogeographic Structures of Wild Radish (Raphanus sativus L. var. raphanistroides Makino) in Japan
Source: PLoS One. 2015 Aug 6;10(8):e0135132. doi: 10.1371/journal.pone.0135132 (PMC4527673; doi:10.1371/journal.pone.0135132)

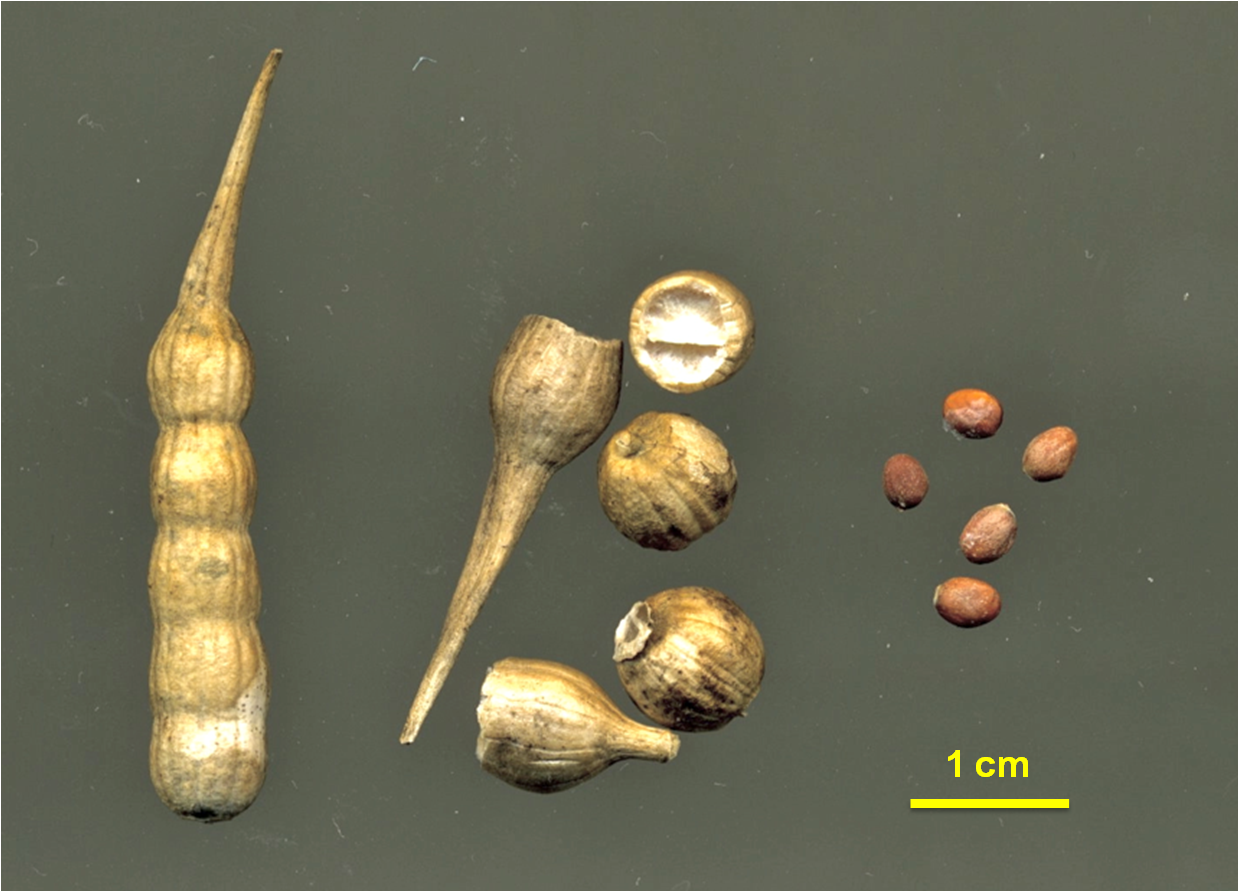

Supplement: S1 Fig — Fruits of wild radish are capsules (left), however, its spongy pericarp is indehiscent and separate at each locule. Seawater dispersal is usually accomplished in this unit (middle). One seed is enveloped by spongy pericarp in each locule (right). (TIF) [file pone.0135132.s001.tif]

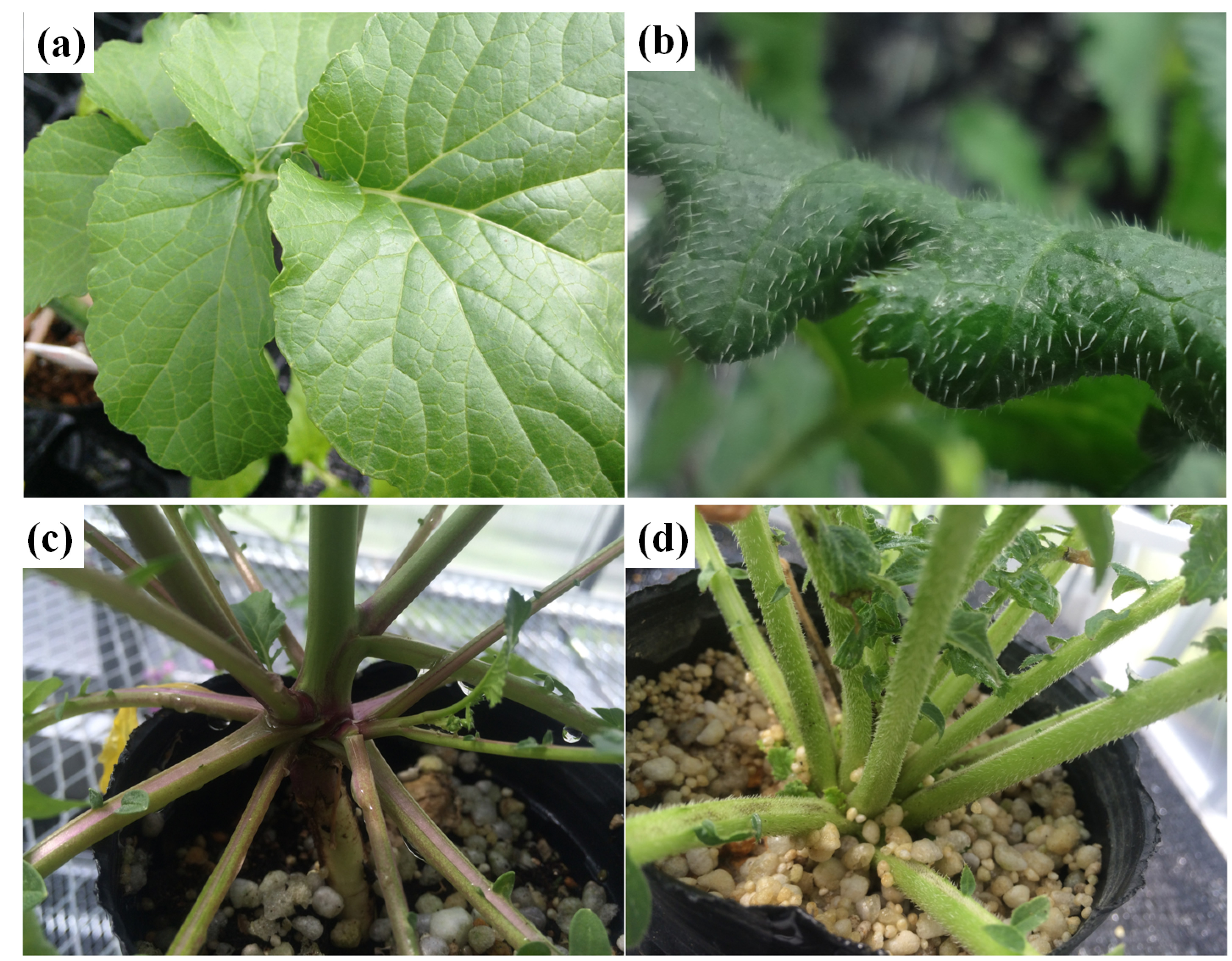

Supplement: S2 Fig — (a) Leaf of wild radish from the Ryukyu Islands; (b) Leaf of wild radish from mainland Japan; (c) Stem of wild radish from the Ryukyu Islands; (d) Stem of wild radish from mainland Japan. Wild radish were planted under identical cultivation conditions (with and without vernalization) in a greenhouse (21°C). (TIF) [file pone.0135132.s002.tif]

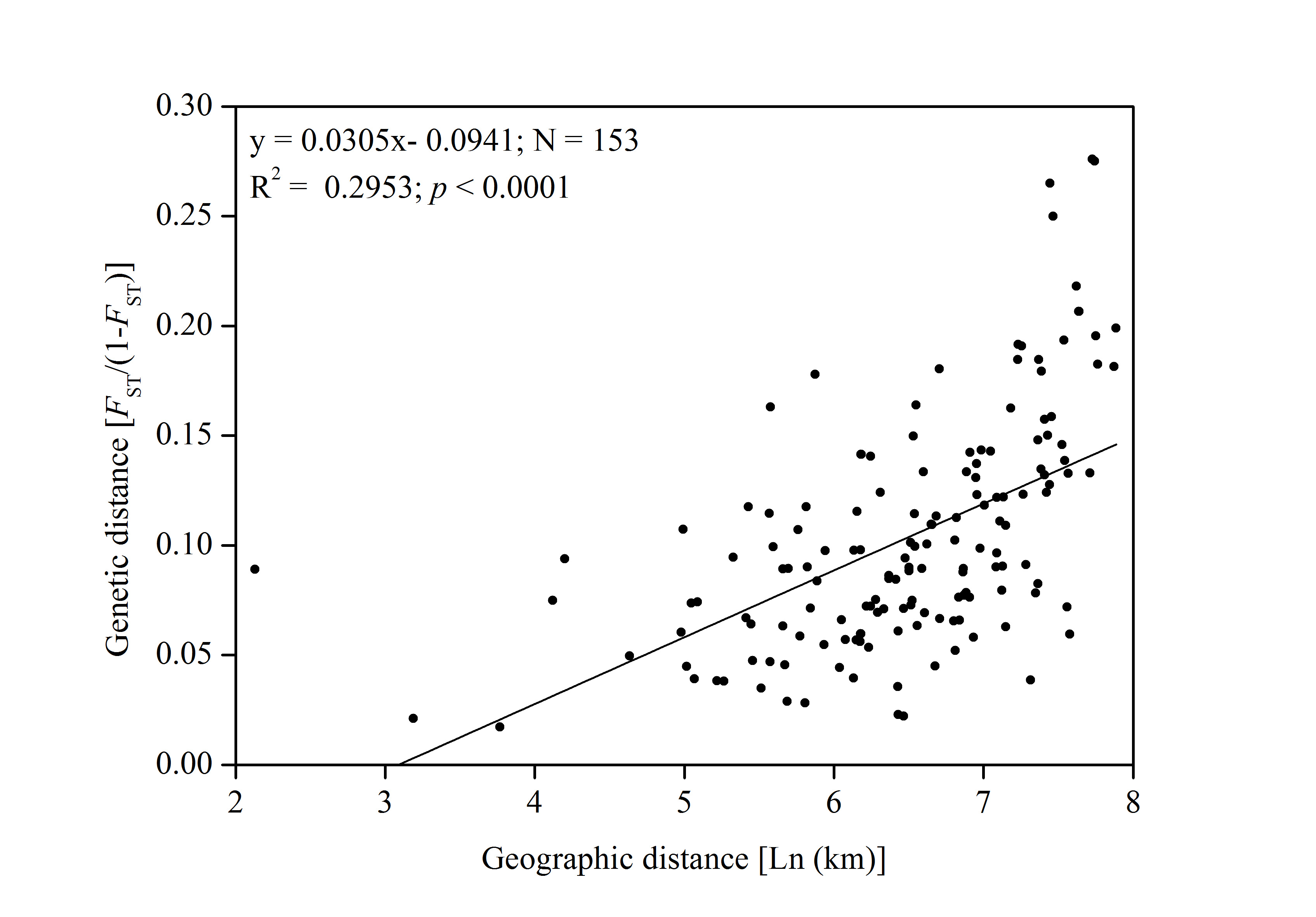

Supplement: S3 Fig — (TIF) [file pone.0135132.s003.tif]

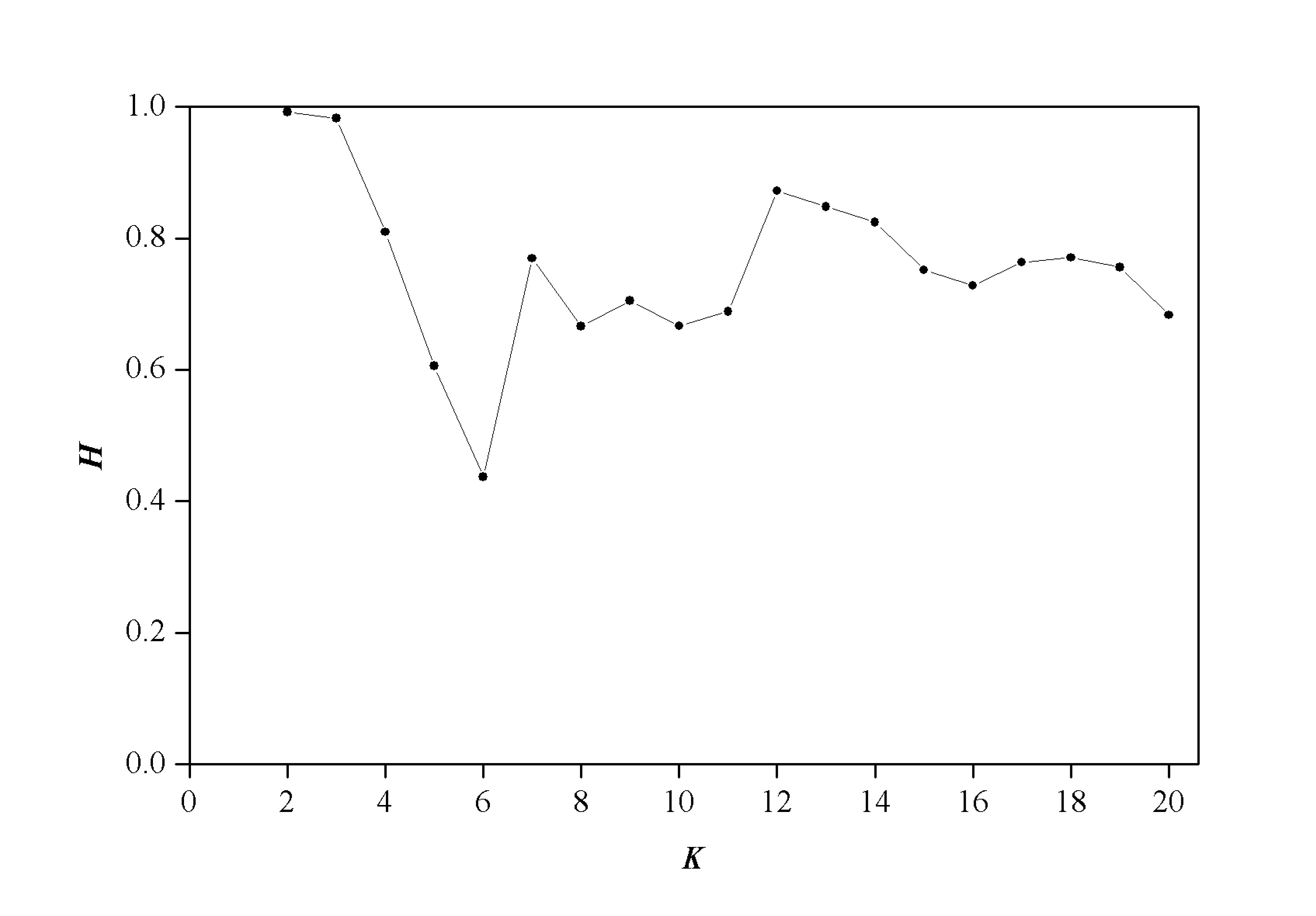

Supplement: S4 Fig — (TIF) [file pone.0135132.s004.tif]

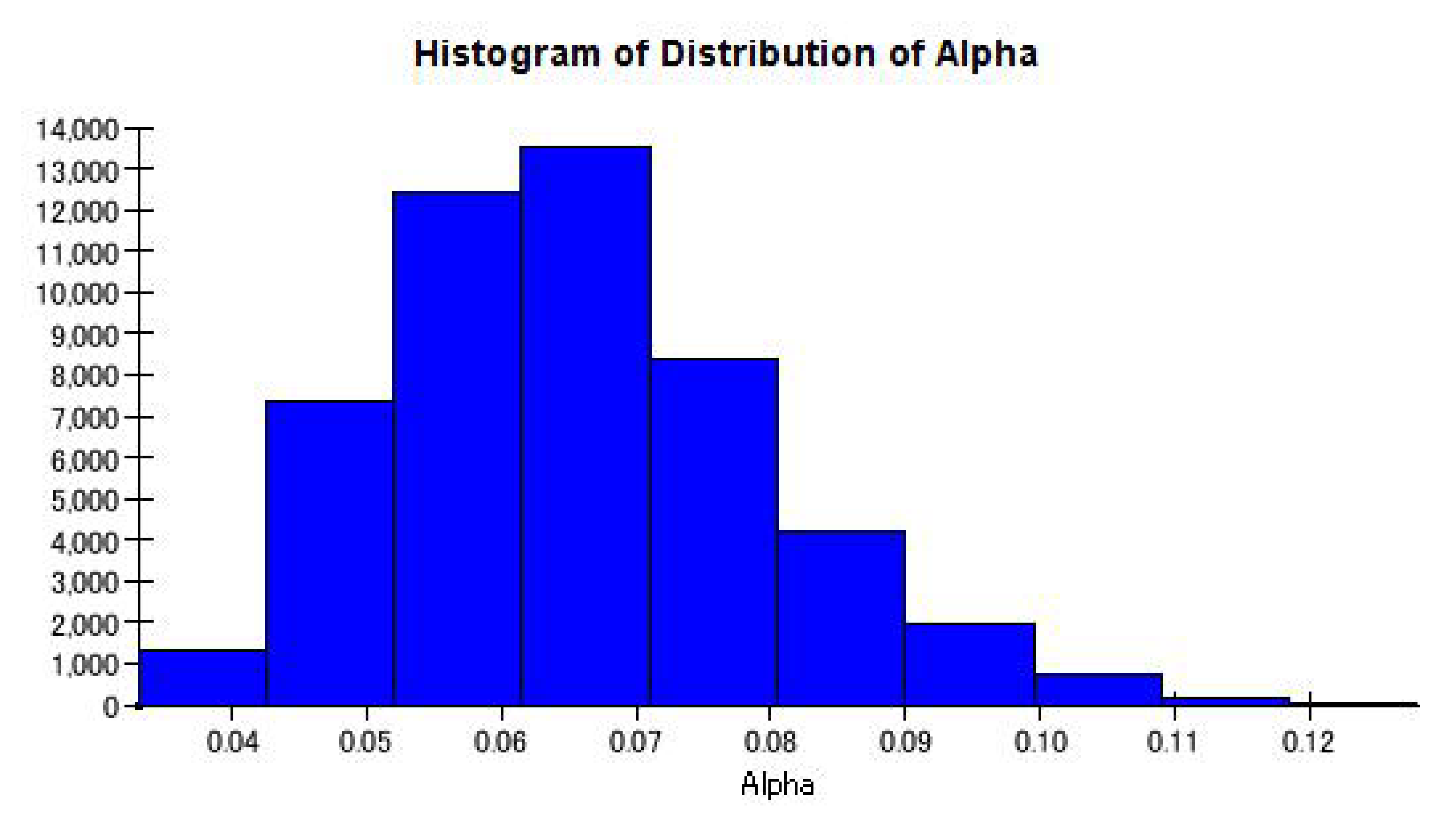

Supplement: S5 Fig — (TIF) [file pone.0135132.s005.tif]

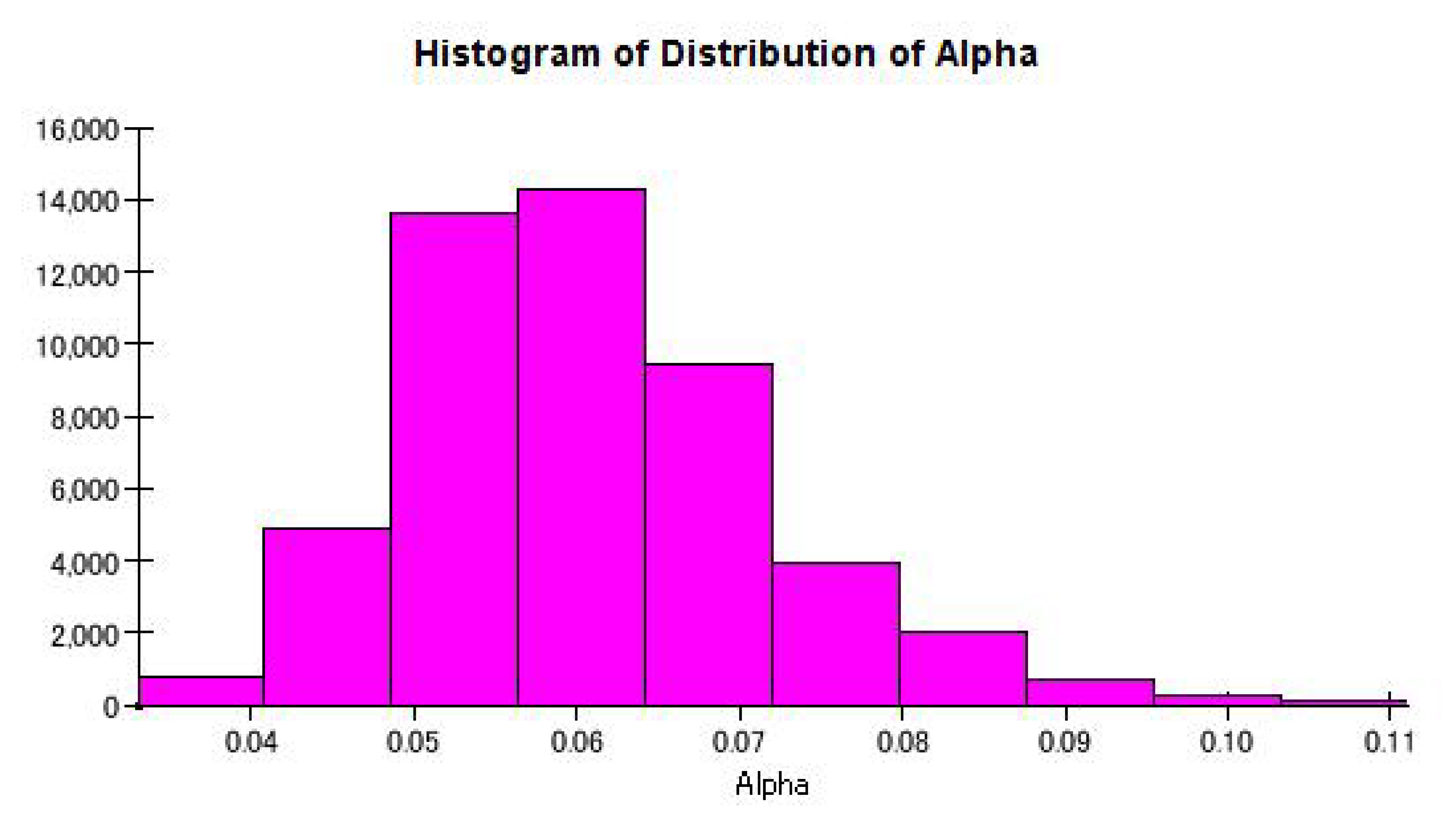

Supplement: S6 Fig — (TIF) [file pone.0135132.s006.tif]

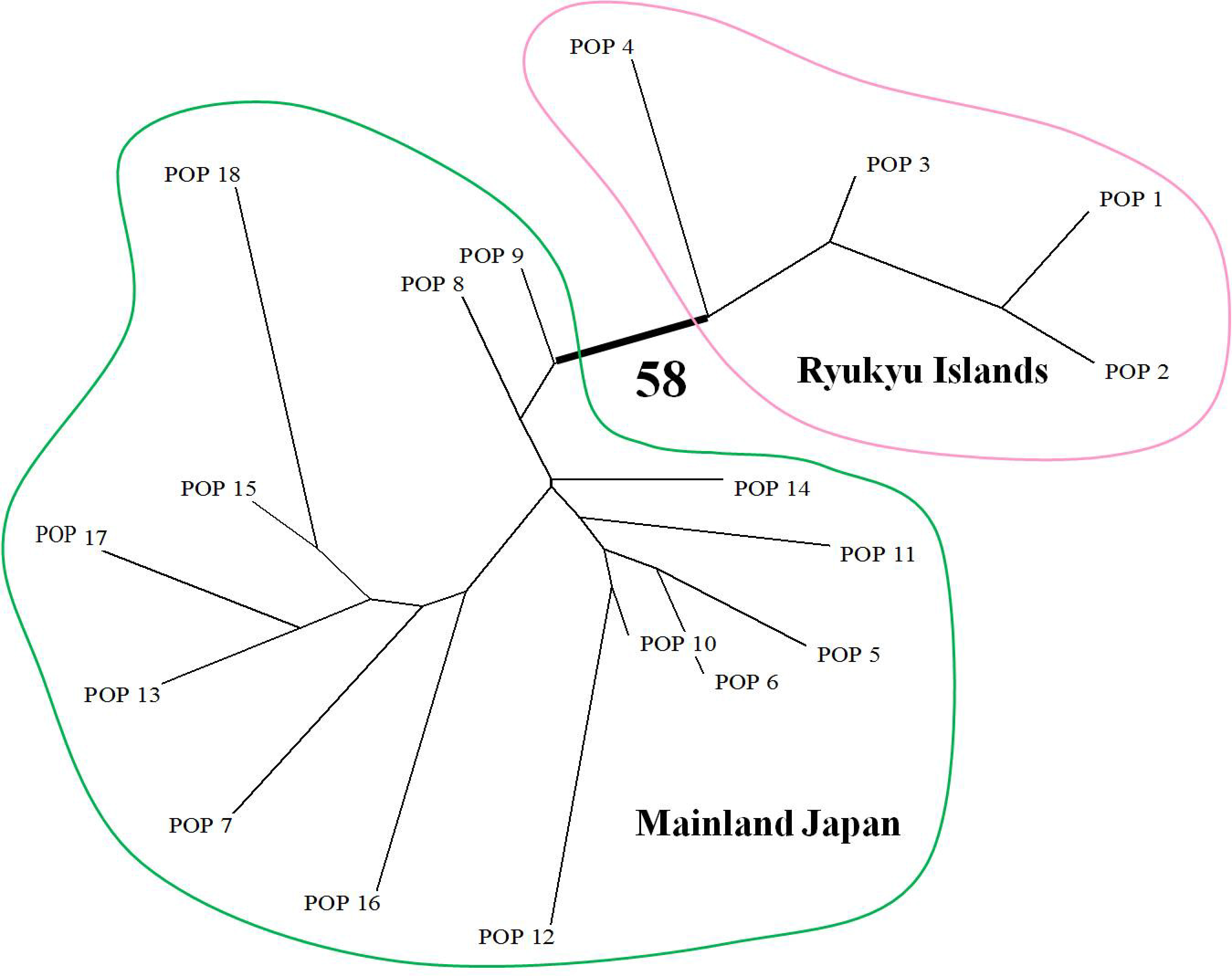

Supplement: S7 Fig — (TIF) [file pone.0135132.s007.tif]
