# Supplementary material for: Distinct Phylogeographic Structures of Wild Radish (Raphanus sativus L. var. raphanistroides Makino) in Japan
Source: PLoS One. 2015 Aug 6;10(8):e0135132. doi: 10.1371/journal.pone.0135132 (PMC4527673; doi:10.1371/journal.pone.0135132)
Supplement: S1 Table — (DOCX) [file pone.0135132.s008.docx]

**S1 Table. Nine nSSR loci used for this study**

| Locus | Primer sequence (5′-3′) | Repeat motif | Size range  (bp) | *H*_O_ | *H*_E_ | *N*a | Dev. from HWE* | GenBank accession no. | References |
| --- | --- | --- | --- | --- | --- | --- | --- | --- | --- |
| Rm19 | F: GCAACCACTATCAAACTCCGTTAT | (AC)_9_ | 295-305 | 0.671 | 0.741 | 0.091 | *p* < 0.05 | AB506684 | [27] |
|  | R: GGGGAAAATACTAAGATGGGTGT |  |  |  |  |  |  |  |  |
| REL-13 | F: CTAGCAATGCATACCAAACAG | (CA)_13_ | 148-166 | 0.263 | 0.500 | 0.310 | *p* < 0.05 | AB630376 | [28] |
|  | R: AACTTGGTCGTTGAGCAG |  |  |  |  |  |  |  |  |
| REL-16 | F: ACAGCAACGTTTTCAAGTGCTC | (TG)_13_ | 183-203 | 0.352 | 0.452 | 0.124 | *p* < 0.05 | AB630377 | [28] |
|  | R: CTCACATGCAATGCAATGCATAC |  |  |  |  |  |  |  |  |
| RsSA014 | F: AATAAGCATGTGGTGGGAAGTTA | (GA)_11_ | 171-183 | 0.298 | 0.648 | 0.370 | *p* < 0.05 | AB608424 | [29] |
|  | R: GGGTTTATGAAAGGGATTTTGTC |  |  |  |  |  |  |  |  |
| RsHR026 | F: AAGCGTGTCATCAGATCCCAGA | (GA)_13_ | 117-137 | 0.296 | 0.689 | 0.398 | 0.1586 | AB608635 | [29] |
|  | R: CATTCTCTCAATGCATAAGATTGAGC |  |  |  |  |  |  |  |  |
| RsSH048 | F: TCGTCCGTTATGTATGTTACTCTCA | (GT)_11_ | 193-197 | 0.317 | 0.688 | 0.369 | *p* < 0.05 | AB608499 | [29] |
|  | R: TATGCGTACTCCGTAAGACAATGTA |  |  |  |  |  |  |  |  |
| RsSA078 | F: AAATGCATCCTAAATGATAAAGTC | (GA)_20_ | 124-180 | 0.755 | 0.897 | 0.086 | *p* < 0.05 | AB608454 | [29] |
|  | R: AGAATCGGATCTAAAGGCGATAA |  |  |  |  |  |  |  |  |
| RsSA083 | F: GCAATGGTTACAAGACAAGGTTTTA | (CT)_18_ | 144-166 | 0.547 | 0.767 | 0.167 | *p* < 0.05 | AB608458 | [29] |
|  | R: CTTCAGATTATTTGCAGCAGCATC |  |  |  |  |  |  |  |  |
| RsSA085 | F: GTTATGAGTTTCTGTGGAAAGTTCG | (GA)_15_ | 142-170 | 0.632 | 0.714 | 0.061 | 0.5688 | AB608460 | [29] |
|  | R: GACTTTTCTTTGTCGACTGTTCTTC |  |  |  |  |  |  |  |  |
| Mean |  |  |  | 0.453 | 0.677 | 0.220 |  |  |  |

*H*_E_, expected heterozygosity; *H*_O_, observed heterozygosity; *N*a, the frequency of null alleles, *N*a= (*H*_E_-*H*_O_)/ (*H*_E_+*H*_O_).

**P* value for HWE test.
