# Supplementary material for: Distinct Phylogeographic Structures of Wild Radish (Raphanus sativus L. var. raphanistroides Makino) in Japan
Source: PLoS One. 2015 Aug 6;10(8):e0135132. doi: 10.1371/journal.pone.0135132 (PMC4527673; doi:10.1371/journal.pone.0135132)
Supplement: S2 Table — (DOCX) [file pone.0135132.s009.docx]

| Region | Primer name | Sequence 5′-3′ | Length (bp) | T_m_/ ºC | References |
| --- | --- | --- | --- | --- | --- |
| *trn*L-F | trnL(e) | GGTTCAAGTCCCTCTATCCC | 385 | 54 | [51] |
|  | trn (f) | ATTTGAACTGGTGACACGAG |  |  |  |
| *trn*T-L | trnT(a)-rs | GTTCTAACCTCATGCGA | 492 | 58 | [51] |
|  | trnL(b) | TCTACCGATTTCGCCATATC |  |  |  |
| *rpl*20-*rps*12 | rpl20-rs | TTCCAATGGCTTTTGCTACTC | 498 | 58 | [52] |
|  | 5′-rps12-rs | GATAGATCGTTCATATCTTC |  |  |  |
