# Supplementary material for: Distinct Phylogeographic Structures of Wild Radish (Raphanus sativus L. var. raphanistroides Makino) in Japan
Source: PLoS One. 2015 Aug 6;10(8):e0135132. doi: 10.1371/journal.pone.0135132 (PMC4527673; doi:10.1371/journal.pone.0135132)
Supplement: S3 Table — (DOCX) [file pone.0135132.s010.docx]

**S3 Table. Probability of a Bottleneck effect for each of the 18 populations of wild radish**

| Pop. | SIGN TEST | | WILCOXON TEST | |
| --- | --- | --- | --- | --- |
|  | IAM | SMM | IAM | SMM |
| 1 | 0.00332** | 0.18546 | 0.00195** | 0.25000 |
| 2 | 0.00250** | 0.15476 | 0.00195** | 0.01953* |
| 3 | 0.00263** | 0.17563 | 0.00195** | 0.00977** |
| 4 | 0.00179** | 0.00424** | 0.00195** | 0.00195** |
| 5 | 0.00250** | 0.61941 | 0.00195** | 0.42578 |
| 6 | 0.00302** | 0.41680 | 0.00195** | 0.30078 |
| 7 | 0.00211** | 0.03814* | 0.00195** | 0.00586** |
| 8 | 0.02155* | 0.34427 | 0.00391** | 0.25000 |
| 9 | 0.02959* | 0.40540 | 0.00391** | 0.49609 |
| 10 | 0.00209** | 0.16703 | 0.00195** | 0.20313 |
| 11 | 0.00158** | 0.03478* | 0.00195** | 0.00586** |
| 12 | 0.00212** | 0.00523** | 0.00195** | 0.00195** |
| 13 | 0.00200** | 0.39464 | 0.00195** | 0.04883* |
| 14 | 0.02410* | 0.17494 | 0.00391** | 0.30078 |
| 15 | 0.00246** | 0.61909 | 0.00195** | 0.30078 |
| 16 | 0.00170** | 0.03188* | 0.00195** | 0.01367** |
| 17 | 0.00260** | 0.40332 | 0.00195** | 0.16406 |
| 18 | 0.00279** | 0.17517 | 0.00195** | 0.04883* |

IAM: infinite allele mutation model; SMM: stepwise mutation model;

One asterisk: *p* < 0.05; Double asterisk: *p* < 0.01.
